# Lonidamine potentiates the oncolytic efficiency of M1 virus independent of hexokinase 2 but via inhibition of antiviral immunity

**DOI:** 10.1186/s12935-020-01598-w

**Published:** 2020-11-02

**Authors:** Jing Cai, Wenbo Zhu, Yuan Lin, Jun Hu, Xincheng Liu, Wencang Xu, Ying Liu, Cheng Hu, Songmin He, Shoufang Gong, Guangmei Yan, Jiankai Liang

**Affiliations:** 1 Department of Pharmacology, Zhongshan School of Medicine, Sun Yat-Sen University, 74 Zhongshan Road II, Guangzhou, 510080 China; 2 Guangzhou Virotech Pharmaceutical Co., Ltd., Guangzhou, 510663 China; 3 Department of Infectious Diseases, The Third Affiliated Hospital of Sun Yat-Sen University, Guangzhou, 510630 China; 4 Department of Urology, The Third Affiliated Hospital of Sun Yat-Sen University, Guangzhou, 510630 China

**Keywords:** Oncolytic virus, M1 virus, Lonidamine, Glycolysis, Colorectal carcinoma, Antiviral immunity

## Abstract

**Background:**

Viruses are obligate parasites that depend on host cells to provide the energy and molecular precursors necessary for successful infection. The main component of virus-induced metabolic reprogramming is the activation of glycolysis, which provides biomolecular resources for viral replication. However, little is known about the crosstalk between oncolytic viruses and host glycolytic processes.

**Methods:**

A MTT assay was used to detect M1 virus-induced cell killing. Flow cytometry was used to monitor infection of M1 virus expressing the GFP reporter gene. qPCR and western blotting were used to detect gene expression. RNA sequencing was performed to evaluate gene expression under different drug treatments. Scanning electron microscopy was performed to visualize the endoplasmic reticulum (ER). Caspase activity was detected. Last, a mouse xenograft model was established to evaluate the antitumor effect in vivo. Most data were analyzed with a two-tailed Student’s t test or one-way ANOVA with Dunnett’s test for pairwise comparisons. Tumor volumes were analyzed by repeated measures of ANOVA. The Wilcoxon signed-rank test was used to compare nonnormally distributed data.

**Results:**

Here, we showed that the glucose analog 2-deoxy-d-glucose (2-DG) inhibited infection by M1 virus, which we identified as a novel type of oncolytic virus, and decreased its oncolytic effect, indicating the dependence of M1 replication on glycolysis. In contrast, lonidamine, a reported hexokinase 2 (HK2) inhibitor, enhanced the infection and oncolytic effect of M1 virus independent of HK2. Further transcriptomic analysis revealed that downregulation of the antiviral immune response contributes to the lonidamine-mediated potentiation of the infection and oncolytic effect of M1 virus, and that MYC is the key factor in the pool of antiviral immune response factors inhibited by lonidamine. Moreover, lonidamine potentiated the irreversible ER stress-mediated apoptosis induced by M1 virus. Enhancement of M1′s oncolytic effect by lonidamine was also identified in vivo.

**Conclusions:**

This research demonstrated the dependence of M1 virus on glycolysis and identified a candidate synergist for M1 virotherapy.

## Background

Oncolytic viruses are natural or genetically engineered organisms, and they constitute an ideal therapeutic platform for treating tumor patients on the basis of their ability to selectively self-replicate in and kill tumor cells but leave normal cells intact [1, 2]. The antitumor mechanisms of oncolytic viruses include direct cell lysis and modulation of the tumor microenvironment (TME). Selective replication of oncolytic viruses in tumor cells induces immunogenic cell death, resulting in the release of antigens and danger-associated molecular patterns (DAMPs) that subsequently activate both innate and adaptive immune responses, converting immunologically “cold” tumors to “hot” ones that harbor high levels of tumor-infiltrating lymphocytes, tumor antigens and mutational burden. The heating of “cold” tumors by oncolytic viruses makes these viruses an ideal platform to combine with immune checkpoint inhibitors, which target immunologically “hot” tumors [3]. To date, only one oncolytic virus has been approved in the United States and Europe—talimogene laherparepvec (T-VEC), derived from herpes simplex virus type 1 (HSV-1) [4]. Numerous clinical trials are underway with oncolytic virus monotherapy or combination therapy with other antitumor drugs [5].

M1 is a strain of alphavirus that has an 11.7 kb positive-sense single-stranded RNA genome and was isolated from a group of Culex mosquitoes [6]. Previously, we identified M1 virus as a novel candidate oncolytic virus that selectively targets zinc finger antiviral protein (ZAP)-deficient tumors [7]. No toxicity was observed after multiple rounds of repeated intravenous injection of M1 virus in nonhuman primates, suggesting the safety of this virus for future use in cancer patients [8]. Moreover, some clinically approved anticancer drugs, such as VCP inhibitors [9], DNA-PK inhibitors [10], Smac mimetics [9], and BCL-XL inhibitors [11], can potentiate the oncolytic effect of M1 virus in various types of cancers. These studies support M1 as a promising oncolytic virus in clinical cancer therapy.

Viruses have evolved mechanisms to usurp the host’s metabolic resources, hijack and control the cellular translational and transcriptional machinery to synthesize and assemble new virions [2]. Even when ample oxygen is present, a wide variety of viruses can activate glycolysis, which derives the energy and carbon production necessary for the synthesis of both cellular biomolecules and viral particles [12], mirroring the Warburg effect in cancer [13]. Both DNA and RNA viruses, including adenovirus [14], Epstein-Barr virus (EBV) [15], hepatitis C virus (HCV) [16], dengue virus [17] etc., can induce glycolytic flux. The multiple specific viral mechanisms enhancing glycolysis are beginning to be elucidated. Among these mechanisms, upregulation and activation of hexokinase 2 (HK2) [16, 17], the first rate-limiting enzyme of glycolysis, is a key event. Virally induced metabolic reprogramming can clearly substantially impact infection outcomes, highlighting the promise of targeting these processes for the development of antiviral therapeutics [12]. Although recent studies have characterized virus-induced changes in glycolysis, the crosstalk between oncolytic viruses and the host glycolysis process remains poorly understood. Elucidation of this crosstalk is important for developing drugs potentially controlling the risks of oncolytic virotherapy and guiding clinical tumor treatment strategies involving the combination of oncolytic viruses with antitumor drugs targeting the Warburg effect.

In the present study, we utilized two glycolysis inhibitors, the glucose analog 2-deoxy-d-glucose (2-DG) and the reported HK2 inhibitor lonidamine [18]. We found that 2-DG suppressed but lonidamine enhanced the infection and oncolytic effect of M1 virus. However, the promotive effect of lonidamine on M1 virus was independent of HK2, indicating the multiple targets of lonidamine. Further transcriptomic analysis revealed that downregulation of the antiviral immune response contributes to enhancement of M1 virus replication by lonidamine and showed that MYC is the key factor among the pool of antiviral immune response factors. Lonidamine potentiates the irreversible endoplasmic reticulum (ER) stress-mediated apoptosis induced by M1 virus. This research identified a candidate synergist for M1 virotherapy.

## Methods

### Cell culture and M1 viruses

HCT 116 (RRID:CVCL_0291) and HCT-8 (RRID:CVCL_2514) cell lines were from American Type Culture Collection (ATCC). Cells were cultured in DMEM supplemented with 10% (vol/vol) FBS (Thermo Fisher Scientific) and 1% penicillin/streptomycin (Thermo Fisher Scientific). All cell lines were cultured at 37 °C in a 5% CO_2_ environment. All cell lines were authenticated by GENWIZ, Inc. China using the GenePrint 10 System (Promega) and were mycoplasma free according to the MycoGuard mycoplasma PCR detection kit (MPD-T-050, GeneCopoeia).

The M1 virus was grown in the Vero cell line and collected for experiments. The M1 virus was provided by Guangzhou Virotech Pharmaceutical Technology Co., Ltd. M1-GFP is a recombinant M1 engineered to express jellyfish green fluorescent protein [10].

### Cell viability assay

Cells were seeded in 96-well plates at 3000 cells per well. After different treatments indicated in the figure legends were administered, 3-(4,5-dimethylthiazol-2-yl)-2,5-diphenyltetrazolium bromide (MTT) was added (1 mg/ml) and incubated at 37 °C for 3 h. The supernatants were removed, and the MTT precipitate was dissolved in 100 μl of DMSO. The optical absorbance was determined at 570 nm by a microplate reader (synergy H1, Gene Company).

### Antibodies and reagents

The following antibodies and reagents were used in this study: HK2 (#2867S, Cell Signaling Technology, RRID: AB_2232946); Ki-67 (#9449, Cell Signaling Technology, RRID: AB_2797703); MYC (#MA1-980, ThermoFisher Scientific, RRID: AB_558470); SECTM1 (#PA5-42725, ThermoFisher Scientific, RRID: AB_2606358); ADAM11 (#PA5-50593, ThermoFisher Scientific, RRID: AB_2636046); 2-DG (#S4701, Selleckchem); Lonidamine (#S2610, Selleckchem). Human IFN-α High sensitivity ELISA kit (EK199HS-01, MultiSciences). Human IFN-β ELISA kit (EK1236-01, MultiSciences).

### RNA-sequencing

HCT 116 tumor cells were treated with control, M1 (MOI = 1 pfu/cell), Lonidamine (50 μM) or M1 (MOI = 1 pfu/cell) plus Lonidamine (50 μM) for 24 h. Total RNA was extracted from 1 × 10^6^ cells with TRIzol Reagent (Thermo Fisher Scientific) and was sent to HaploX Genomics Center (HGC, China) for RNA sequencing by Illumina platform.

### RNA interference

siRNAs specific to different genes and control nontargeting siRNA were synthesized by Sigma-Aldrich. The cells were transfected with the siRNAs (50 nM) using Lipofectamine RNAiMAX (Thermo Fisher Scientific) in Opti-MEM medium (Thermo Fisher Scientific). The sequences of the siRNAs are listed below.

- si-HK2 001: 5′-CTGTGAAGTTGGCCTCATT-3’
- si-HK2 002: 5′-ACGACAGCATCATTGTTAA-3’
- si-HK2 003: 5′-CTGGCTAACTTCATGGATA-3’
- si-MYC 001: 5′-GAGGAGACATGGTGAACCA-3’
- si-MYC 002: 5′-GGGTCAAGTTGGACAGTGT-3’
- si-MYC 003: 5′-CGACGAGACCTTCATCAAA-3’
- si-ADAM11 001: 5′-GCTGTAGCATCGACGAGTA-3’
- si-ADAM11 002: 5′-TCCTCTCCTCGCAATACGT-3’
- si-ADAM11 003: 5′-GCAAAGACTGCAGTATCCA-3’
- si-SECTM1 001: 5′-GTGGGACACCAGAGAAATA-3’
- si-SECTM2 002: 5′-TGGTCATGTTCGCCTGGTA-3’
- si-SECTM3 003: 5′-GGCACAGCTGGTGATCAAA-3’.

### RT-qPCR

Total RNA was extracted using TRIzol (Thermo Fisher Scientific), and 2 μg of total RNA was reverse-transcribed to cDNA with oligo (dT) (synthesized by Thermo Fisher Scientific) and RevertAid Reverse Transcriptase (Thermo Fisher Scientific). The expression levels of the specific genes were calculated by the comparative Ct method using SuperReal PreMix SYBR Green (FP204-02, TIANGEN) and an Applied Biosystem 7500 Fast Real-Time PCR system (Thermo Fisher Scientific, RRID: SCR_014596). The sequences of the primers are listed below:

- C10orf10 Forward: 5′-GTGAGGTCTATATCTCGACTGGC-3’.
- C10orf10 Reverse: 5′-ACTGAAACGTGCGGTGATGT-3’.
- UCP2 Forward: 5′-GGAGGTGGTCGGAGATACCAA-3’.
- UCP2 Reverse: 5′-ACAATGGCATTACGAGCAACAT-3’.
- PLEKHA4 Forward: 5′-TTGGCCGCTGACACCTTAG-3’.
- PLEKHA4 Reverse: 5′-GGTTGCCCATAGTCGTCCC-3’.
- SECTM1 Forward: 5′-CGCCATCTTCAATGAGGTGG-3’.
- SECTM1 Reverse: 5′-CCAGCGTGACTTGTCTGTTATT-3’.
- ETV6 Forward: 5′-GCTCAGTGTAGCATTAAGCAGG-3’.
- ETV6 Reverse: 5′-CGAGGAAGCGTAACTCGGC-3’.
- MYC Forward: 5′-GTCAAGAGGCGAACACACAAC-3’.
- MYC Reverse: 5′-TTGGACGGACAGGATGTATGC-3’.
- FKBP5 Forward: 5′-AATGGTGAGGAAACGCCGATG-3’.
- FKBP5 Reverse: 5′-TCGAGGGAATTTTAGGGAGACT-3’.
- IFIT1 Forward: 5′-GCGCTGGGTATGCGATCTC-3’.
- IFIT1 Reverse: 5′-CAGCCTGCCTTAGGGGAAG-3’.
- ADAM11 Forward: 5′-AACCCAGCCGTCTGGTTAG-3’.
- ADAM11 Reverse: 5′-TGGGATGACGAAACTCACCTG-3’.
- SAT1 Forward: 5′-ACCCGTGGATTGGCAAGTTAT-3’.
- SAT1 Reverse: 5′-TGCAACCTGGCTTAGATTCTTC-3’.

### Western blot analysis

Cell samples were prepared using M-PER Mammalian Protein Extraction Reagent (Thermo Fisher Scientific) and then separated by sodium dodecyl sulfate–polyacrylamide gel electrophoresis. The membranes were visualized with a ChemiDoc XRS + System (Bio-Rad) using Immobilon Western Chemiluminescent HRP Substrate (Millipore).

### Caspase activity assay

HCT 116 and HCT-8 cell lines were treated with different drugs as the figure legend indicated, 100 μl of caspase 3/7, 8 and 9 (Promega) reaction buffer was added to the supernatant and incubated for 30 min. The liquids were transferred to a black-bottomed plate, the luminescence was detected by Synergy H1 microplate reader (Gene Company). The values were normalized to cell numbers (MTT assay).

### HK2 activity assay

Hexokinase II Inhibitor Screening Kit (Colorimetric) (abcam, ab211114) was used to detect the activity of HK2. In brief, prepare the hexokinase 2 enzyme solution by adding hexokinase recombinant enzyme solution to the assay buffer and incubate for 5 min at 25 °C. Then prepare the hexokinase substrate mix according to the instruction. Mix the hexokinase 2 enzyme solution and hexokinase substrate with lonidamine or positive HK2 inhibitor (Bromopyruvic Acid) by gentle shaking. Measure absorbance (OD = 450 nm) on a microplate reader (Gene company) immediately in kinetic mode for 5–30 min at 25 °C. When detecting the activity of hexokinase in cells, cell protein lysate is used to replace the human HK2 recombinant protein as HK2 enzyme for the reaction. Four HK isoforms (HK1, HK2, HK3 and HK4) are found in human, all HK isoforms are capable of converting glucose into glucose-6-phosphate. So in the assay with cell protein lysate, we can only determine whether lonidamine can inhibit the activity of total HK, the activity of each specific HK isoform is unable to be distinguished.

### Animal models

The mouse study was approved by the Animal Ethics and Welfare Committee of Sun Yat-sen University, and all experiments were conducted according to the US “Public Health Service Policy on Humane Care and Use of Laboratory Animals”. HCT 116 (1 × 10^6^ cells/mouse) tumor cells were implanted subcutaneously into the hind flanks of 5-week-old 16 g female BALB/c-nu/nu mice (the mice were bought from Nanjing Biomedical Research Institute, China, and housed in an SPF facility with normal temperature and food). After 6 days, tumors were observed (approximately 50 mm^3^). The mice were randomized and grouped to 4 treatment groups: vehicle control, M1 virus (i.v., 2 × 10^6^ pfu per mouse, daily for 5 days), Lonidamine (i.p., 10 mg/kg, daily for 5 days), combination of M1 virus and Lonidamine. The lengths and widths of the tumors were measured every 3 days, and the tumor volume was calculated according to the formula (length × width^2^)/2. At the termination of the experiment, all mice were euthanized by overdose anesthesia, and the tumors were removed and fixed in 4% paraformaldehyde for subsequent immunohistochemistry (IHC) assays. The study was blind.

### Immunohistochemistry (IHC) assay

The expression of Ki-67 in the tumors was assessed by immunohistochemistry (IHC). Briefly, tumor sections were dewaxed in xylene, hydrated in decreasing concentrations of ethanol, immersed in 0.3% H_2_O_2_-methanol for 30 min, washed with phosphate-buffered saline, and probed with monoclonal antibodies or isotype controls at 4 °C overnight. After being washed, the sections were incubated with biotinylated goat anti-rabbit or anti-mouse IgG at room temperature for 2 h. Immunostaining was visualized with streptavidin/peroxidase complex and diaminobenzidine, and sections were counterstained with hematoxylin.

### Statistics

All statistical analyses were performed using GraphPad Prism software 8.0 (RRID: SCR_002798) and SPSS 18.0 software (RRID: SCR_002865). Most of the data were analyzed by a two-tailed Student’s t test or one-way ANOVA with Dunnett’s test for pairwise comparisons. Tumor volumes were analyzed by repeated measures of ANOVA. Phase contrast and fluorescence pictures were taken with a Nikon Eclipse A1 microscope. The IHC staining intensity was analyzed by ImageScope software (ImageScope, RRID: SCR_014311). The Wilcoxon signed-rank test was used to compare non-normally distributed data. Bars show the mean ± SD of three independent repeated experiments. Significant differences were accepted if the p-value was < 0.05.

## Results

### 2-Deoxy-d-glucose (2-DG) inhibits but lonidamine potentiates the oncolytic effect of M1 virus

**Fig. 1.**
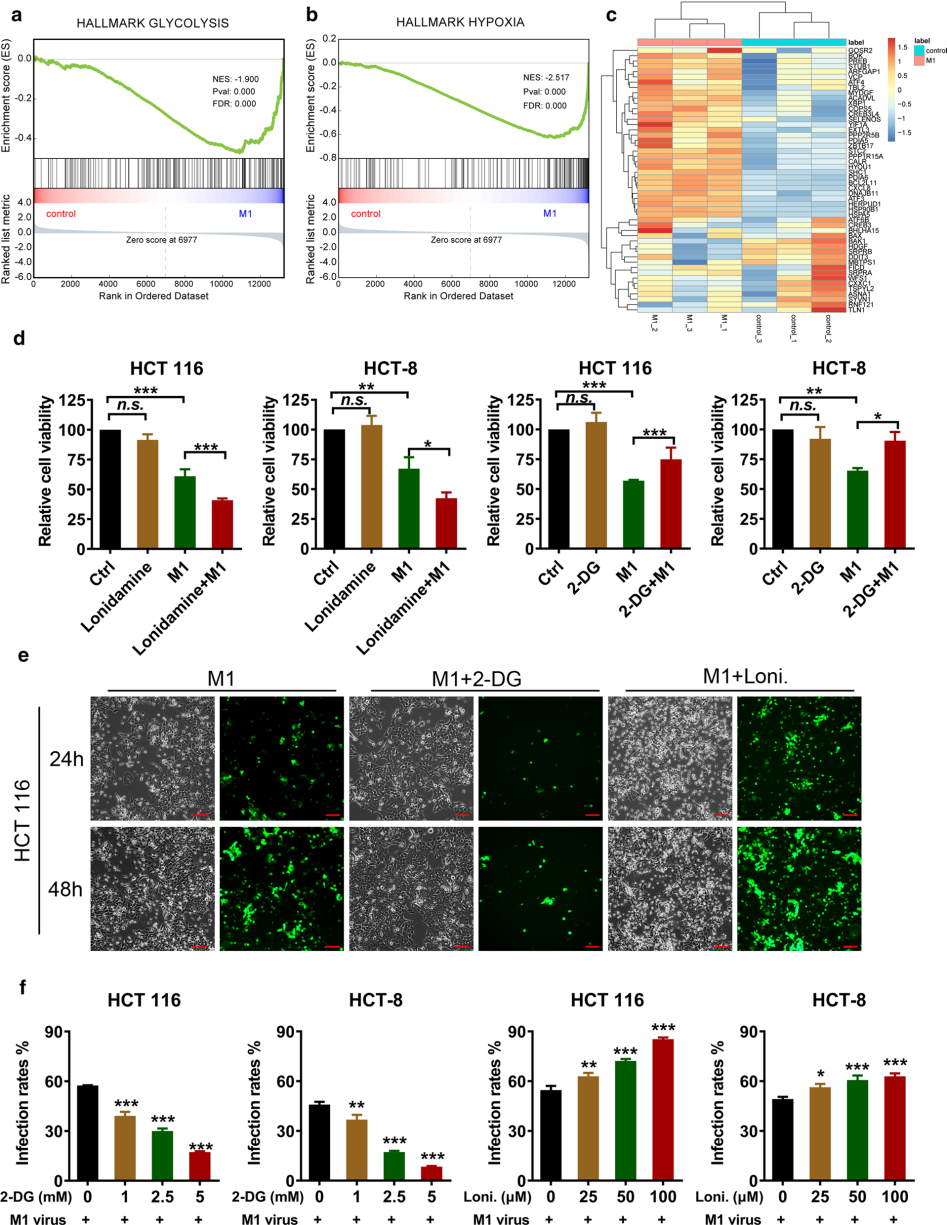
2-DG inhibits but lonidamine potentiates the oncolytic effect of M1 virus. **a**, **b** M1 treatment upregulated glycolysis and hypoxia pathways. The HCT 116 cell line was treated with M1 virus (MOI = 1 pfu/cell) for 24 h, and was then subjected to RNA-seq. GSEA of hallmark glycolysis and hypoxia gene sets is shown. Normalized enrichment score (NES), *p*, and false discovery rate (FDR) values are indicated. **c** Heatmap of gene expression in the glycolysis gene set. **d** The HCT 116 and HCT-8 cell lines were treated with vehicle, 2-DG (2.5 mM), lonidamine (50 μM), M1 virus (MOI = 1 pfu/cell), or M1 virus plus 2-DG/lonidamine for 48 h, and cell viability was detected by MTT. n = 3. Statistical analysis was performed by one-way ANOVA with Dunnett’s tests for pairwise comparisons. **e** The HCT 116 cell line was treated with M1 virus (MOI = 1 pfu/cell) or M1 virus plus 2-DG (2.5 mM)/lonidamine (50 μM) for 24 or 48 h. Phase contrast and fluorescence micrographs are shown. The results shown are one representative result from three similar replicate experiments. Scale bar, 100 μm. **f** The HCT 116 and HCT-8 cell lines were treated with vehicle, 2-DG, lonidamine, M1 virus (MOI = 1 pfu/cell), or M1 virus plus 2-DG/lonidamine for 24 h, and the infection rate of M1 virus (GFP percentage) was determined by flow cytometry. n = 3. Statistical analysis was performed by one-way ANOVA with Dunnett’s tests for pairwise comparisons. The error bars indicate the mean ± SD values from three independent experiments. *ns* nonsignificant; *p < 0.05, **p < 0.01, ***p < 0.001

To explore whether M1 virus induces glycolytic reprogramming, we conducted a transcriptome sequencing in HCT 116 colorectal carcinoma cells upon M1 treatment. Gene set enrichment analysis (GSEA) revealed that M1 treatment upregulated genes in the glycolysis and hypoxia hallmark gene sets (Fig. 1a, b). A heatmap of gene expression in the glycolysis set is shown in Fig. 1c. These findings indicate that consistent with numerous reports of other viruses, M1 virus can activate glycolytic flux.

To further investigate whether M1 virus relies on host cell glycolysis to facilitate its replication, two glycolysis inhibitors—the glucose analog 2-DG and lonidamine, which inhibits hexokinase 2 (HK2), the first rate-limiting enzyme in glycolysis—were used to treat the colorectal carcinoma cell lines HCT 116 and HCT-8. 2-DG inhibited M1 virus-induced cell killing in these cell lines (Fig. 1d), indicating the dependence of M1′s oncolytic effect on host glycolysis. We previously reported that the cancer cell targeting and killing properties of M1 virus depend on the viral replication in cancer cells [7]. We used an M1 virus engineered to express the reporter protein GFP (M1-GFP) to trace viral gene expression and replication in tumor cells. Phase contrast and immunofluorescence microscopy showed that 2-DG suppressed M1 infection and decreased M1-induced cytopathic effects (Fig. 1d, e). Consistent with this result, 2-DG reduced the infection rate of M1 virus, as shown by flow cytometry to determine the percentage of GFP-positive cells (Fig. 1f). These results suggest that the infection and replication of M1 virus in tumor cells are dependent on the glycolysis in the host cells.

Surprisingly, in contrast to 2-DG, another glycolysis inhibitor, lonidamine, potentiated the oncolytic effect and increased the infection rate of M1 virus (Fig. 1d–f), indicating that lonidamine might act synergistically with M1 virus. Exploring the potential mechanism underlying these effects is important.

### Lonidamine enhances M1 virus infection by inhibiting the antiviral immune response independent of HK2

**Fig. 2:**
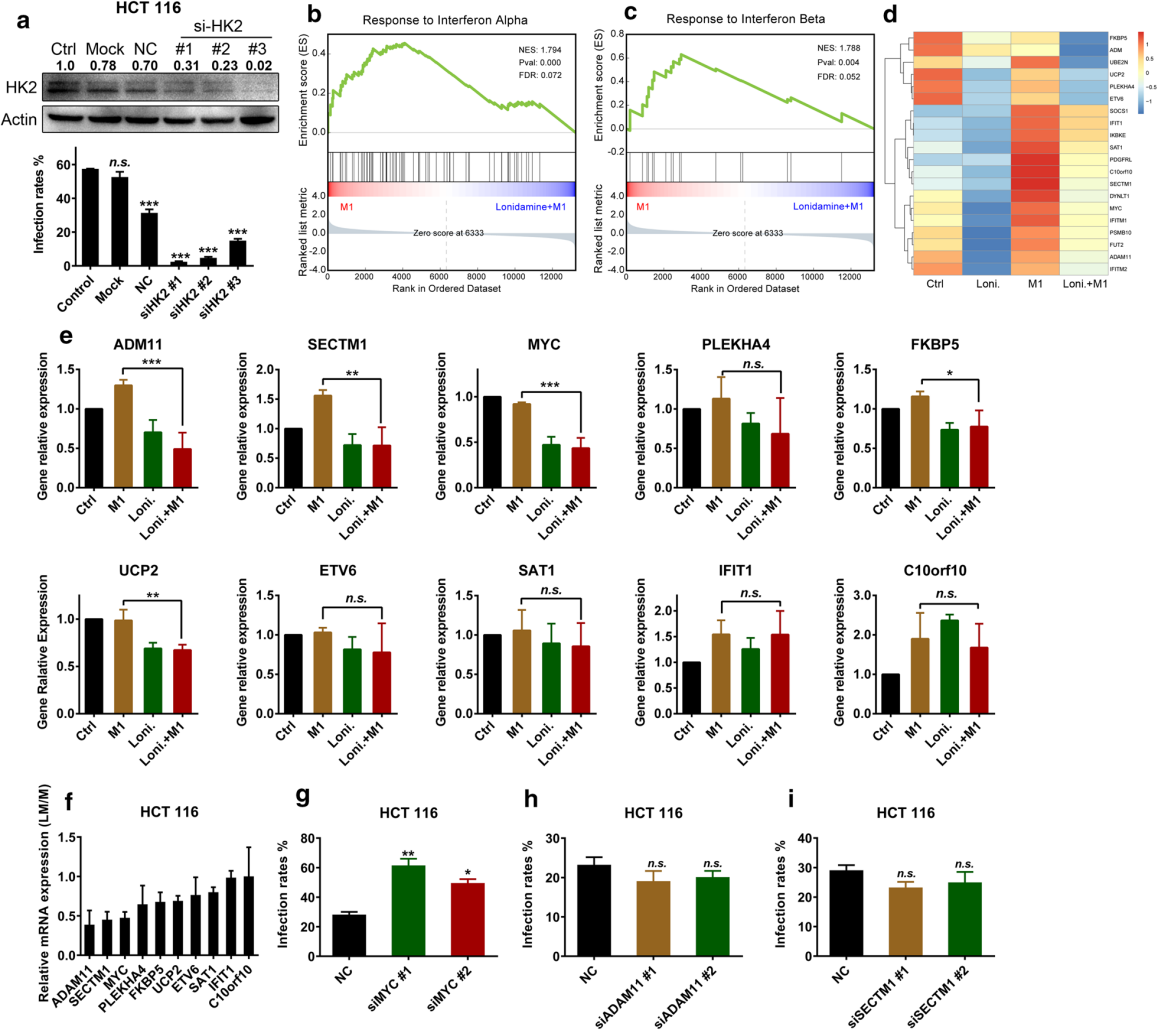
Lonidamine enhances M1 virus infection by inhibiting the antiviral immune response in a manner independent of HK2. **a** The HCT 116 cell line was treated with siRNAs targeting HK2 for 48 h. M1 virus (MOI = 1 pfu/cell) was added for another 24 h, and the infection rate of M1 virus (GFP percentage) was then determined by flow cytometry. n = 3. Statistical analysis was performed by one-way ANOVA with Dunnett’s test for pairwise comparisons. The knockdown efficiency of the siRNAs (48 h) targeting HK2 in the HCT 116 cell line was determined by western blotting. The relative expression levels of HK-2 compared to those in the control group were calculated and are labeled. GSEA of the IFN-α response gene set (**b**) and IFN-β gene set (**c**) in HCT 116 cells after treatment with M1 (MOI = 1 pfu/cell) or M1 (MOI = 1 pfu/cell) plus lonidamine (50 μM) for 24 h. The NES, *p* and FDR values are indicated in each box. **d** Heatmap of the top 20 IRGs inhibited by lonidamine plus M1 compared with M1 (LM/M) in HCT 116 cells. **e** The HCT 116 cell line was treated with control, M1 (MOI = 1 pfu/cell), lonidamine (50 μM) or M1 (MOI = 1 pfu/cell) plus lonidamine (50 μM) for 24 h, and the relative expression levels of the top 10 IRGs (*ADM11*, *SECTM1*, *MYC*, *PLEKHA4*, *FKBP5*, *UCP2*, *ETV6*, *SAT1*, *IFIT1*, and *C10orf10*) noted in (**d**) were determined by qPCR; n = 3. Statistical analysis was performed by one-way ANOVA with Dunnett’s test for pairwise comparisons. **f** Summary of the top 10 IRGs inhibited by the combination of lonidamine plus M1 compared with M1 alone (LM/M) in (**e**, **f**). **g**–**i** The HCT 116 cell line was treated with siRNAs targeting *ADM11*, *SECTM1*, and *MYC* for 48 h. M1 virus (MOI = 1 pfu/cell) was added for another 24 h, and the M1 virus infection rate (GFP percentage) was determined by flow cytometry. n = 3. Statistical analysis was performed by one-way ANOVA with Dunnett’s test for pairwise comparisons. The error bars indicate the mean ± SD values from three independent experiments with three technical replicates. *ns* nonsignificant; *p < 0.05, **p < 0.01, ***p < 0.001

Lonidamine is an HK2 inhibitor [18], and we confirmed that lonidamine not only inhibited the activity of HK2 in vitro but also inhibited the activity of total HK in HCT 116 cells (Additional file 1A, B). To determine whether lonidamine enhances the oncolytic effect of M1 virus by inhibiting HK2, specific small interfering RNAs (siRNAs) for HK2 were transfected into HCT 116 and HCT-8 cells. Interestingly, knockdown of HK2 did not simulate the effect of lonidamine but instead inhibited the M1 virus infection (Fig. 2a and Additional file 2). The results indicate the existence of another mechanism by which lonidamine potentiates the oncolytic effect of M1 virus. On the other hand, knockdown of HK2 reproduced the effects of 2-DG, indicating the dependence of M1′s oncolytic effect on glycolysis.

To explore the mechanism by which lonidamine promotes the M1 virus infection and replication, gene expression profiling was performed in the HCT 116 cell line treated with vehicle, lonidamine, M1 or lonidamine plus M1. Viral infection and replication induce host antiviral pathways, which consist mainly of interferon (IFN) signaling to hijack viral replication. Among the three classes of IFNs, type I IFNs, the most common of which are IFN-α and IFN-β, are essential for mounting a robust host response against viral infection [19]. We hypothesized that lonidamine might inhibit antiviral IFN pathway activity to potentiate the effect of M1 virus. GSEA revealed that lonidamine strongly inhibited both the IFN-α and IFN-β response pathways after M1 virus infection (Fig. 2b, c). To identify the key factors upregulated by lonidamine, we focused on the expression of 317 IFN-regulated genes (IRGs), which have been identified as crucial anti-alphavirus M1 effectors [7]. A heatmap of the top 20 IRGs inhibited by the combination of lonidamine and M1 compared with M1 alone (LM/M) is shown in Fig. 2d. In addition, qPCR was used to verify the expression of the top 10 IRGs in the HCT 116 cell line. The expression of *ADM11*, *SECTM1*, *and MYC* was significantly inhibited in the lonidamine plus M1 treatment group compared with the M1 treatment group (Fig. 2e). We ranked the degree of gene expression change between the lonidamine plus M1 group and the M1 group (LM/M) and found that these three genes had the lowest expression levels (Fig. 2f). Moreover, M1 virus-induced secretion of IFN-β was inhibited by lonidamine, while the production of IFN-α was unchanged (Additional file 3A, B), indicating that lonidamine attenuates the IFN-β production and subsequent antiviral response induced by M1 virus. In addition, lonidamine did not affect IFN-α production but did inhibit the response of the antiviral signaling cascade to IFN-α (Fig. 2b−f). These results indicate that lonidamine attenuates the IFN-mediated innate immune response of cancer cells, which may result in enhancement of the infection, replication and oncolytic effect of M1 virus.

To further identify the specific IRG that inhibits viral infection, we used siRNAs to knock down the three genes identified above (*ADM11*, *SECTM1*, *and MYC*) in the HCT 116 cell line. The siRNAs effectively knocked down the expression of these genes (Additional file 4A–F), and knockdown of *MYC* significantly increased M1 virus infection (Fig. 2g–i), indicating that MYC might be a key factor suppressed by lonidamine signaling to promote M1 virus infection and replication.

### Lonidamine potentiates M1 virus-mediated endoplasmic reticulum (ER) stress-induced apoptosis

**Fig. 3:**
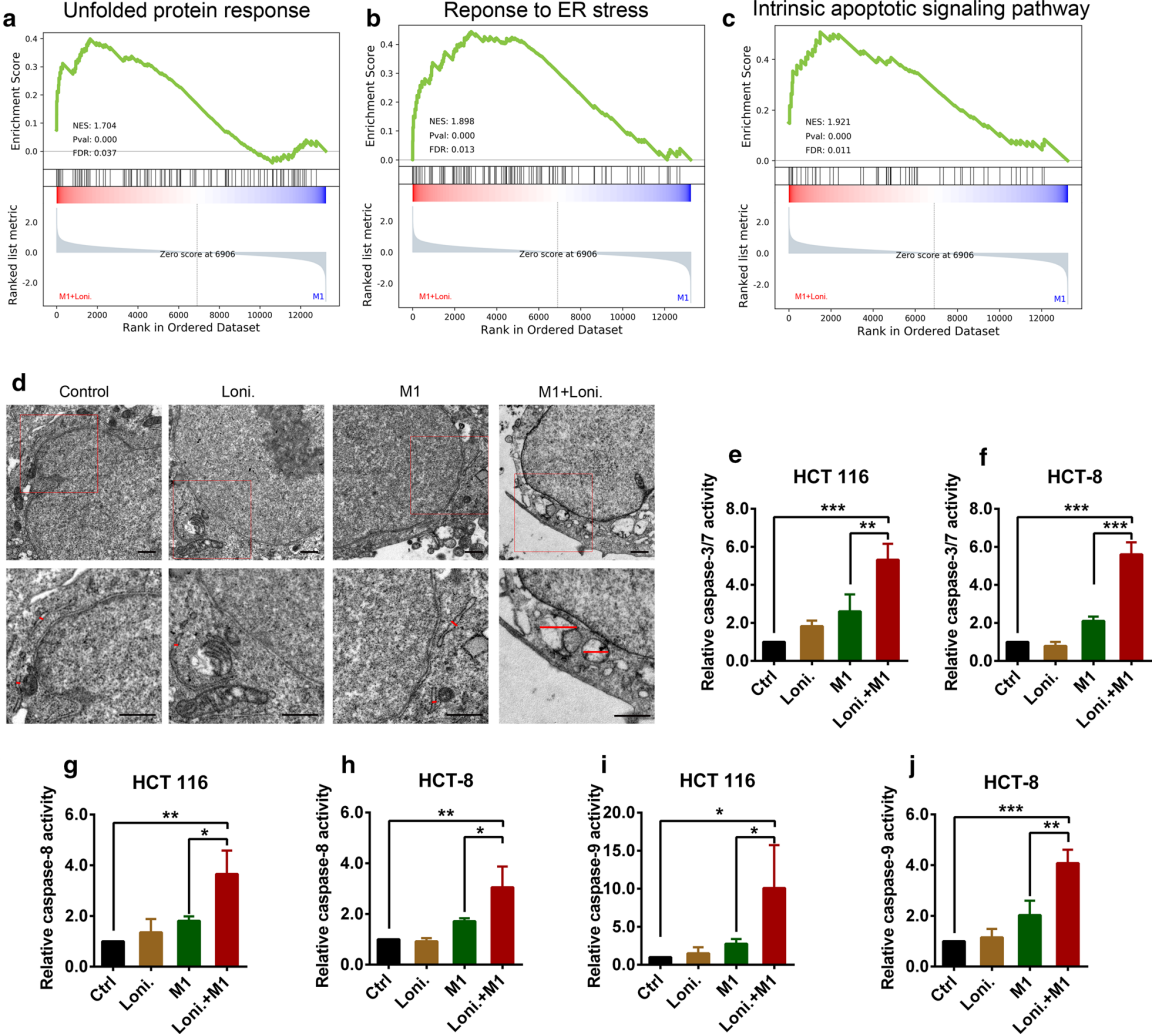
Lonidamine potentiates M1 virus-mediated ER stress-induced apoptosis. **a**–**c** GSEA of the unfolded protein response, response to ER stress, and intrinsic apoptotic signaling pathway gene sets in the HCT 116 cell line after treatment with M1 (MOI = 1 pfu/cell) or M1 (MOI = 1 pfu/cell) plus lonidamine (50 μM) for 24 h. NES, *p* and FDR values are shown in each box. **d** The HCT 116 cell line was treated with vehicle, lonidamine (50 μM), M1 virus (MOI = 1 pfu/cell), or M1 virus plus lonidamine for 24 h, and cellular morphology was observed by SEM. The red lines indicate the diameter of the ER. Scale bars, 1 μm. **e**–**j** The HCT 116 and HCT-8 cell lines were treated with vehicle, lonidamine (50 μM), M1 virus (MOI = 1 pfu/cell), or lonidamine plus M1 virus for 48 h, and caspase-8, caspase-9 and caspase-3/7 activity was assessed. n = 3. Statistical analysis was performed by one-way ANOVA with Dunnett’s tests for pairwise comparisons. The error bars indicate the mean ± SD values from three independent experiments with three technical replicates. *ns* nonsignificant; *p < 0.05, **p < 0.01, ***p < 0.001

We next explored the mechanism by which lonidamine potentiates M1-induced tumor cell killing. Increased viral replication induces aggregation of viral proteins in host cells, which in turn induces the unfolded protein response and changes in the ER, indicating ER stress [20]. Previously, we found that the direct killing effect of M1 virus is mediated by ER stress [7]. Therefore, we hypothesized that lonidamine might enhance the tumor cell killing effect of M1 virus through intense ER stress. GSEA showed that gene sets in the unfolded protein response, response to ER stress, and intrinsic apoptotic signaling pathway were significantly upregulated by treatment with lonidamine plus M1 compared with M1 alone (Fig. 3a–c). Next, scanning electron microscopy (SEM) was used to observe the morphology of the ER in cells. Lonidamine monotherapy did not change the ER structure. Treatment with M1 virus alone induced minimal and slight expansion of the ER, while treatment with the combination of lonidamine and M1 virus induced severe ER expansion in HCT 116 cells (Fig. 3d). The downstream caspase cascades in each treatment group were monitored by assessing the activity of caspase-8, caspase-9 and apoptotic executioner caspase-3/7. Lonidamine monotherapy did not change the activities of caspase-8, caspase-9 and caspase-3/7. Treatment with M1 alone slightly induced caspase-8, caspase-9 and caspase-3/7 activity; however, the activity of these caspases was dramatically elevated after treatment with the combination of M1 and lonidamine (Fig. 3e–j). Taken together, these results suggest that the combination of lonidamine and M1 virus induces irreversible ER stress and apoptosis via caspase-8, caspase-9 and caspase-3/7.

### Lonidamine synergizes with M1 virus to induce tumor regression in vivo

**Fig. 4:**
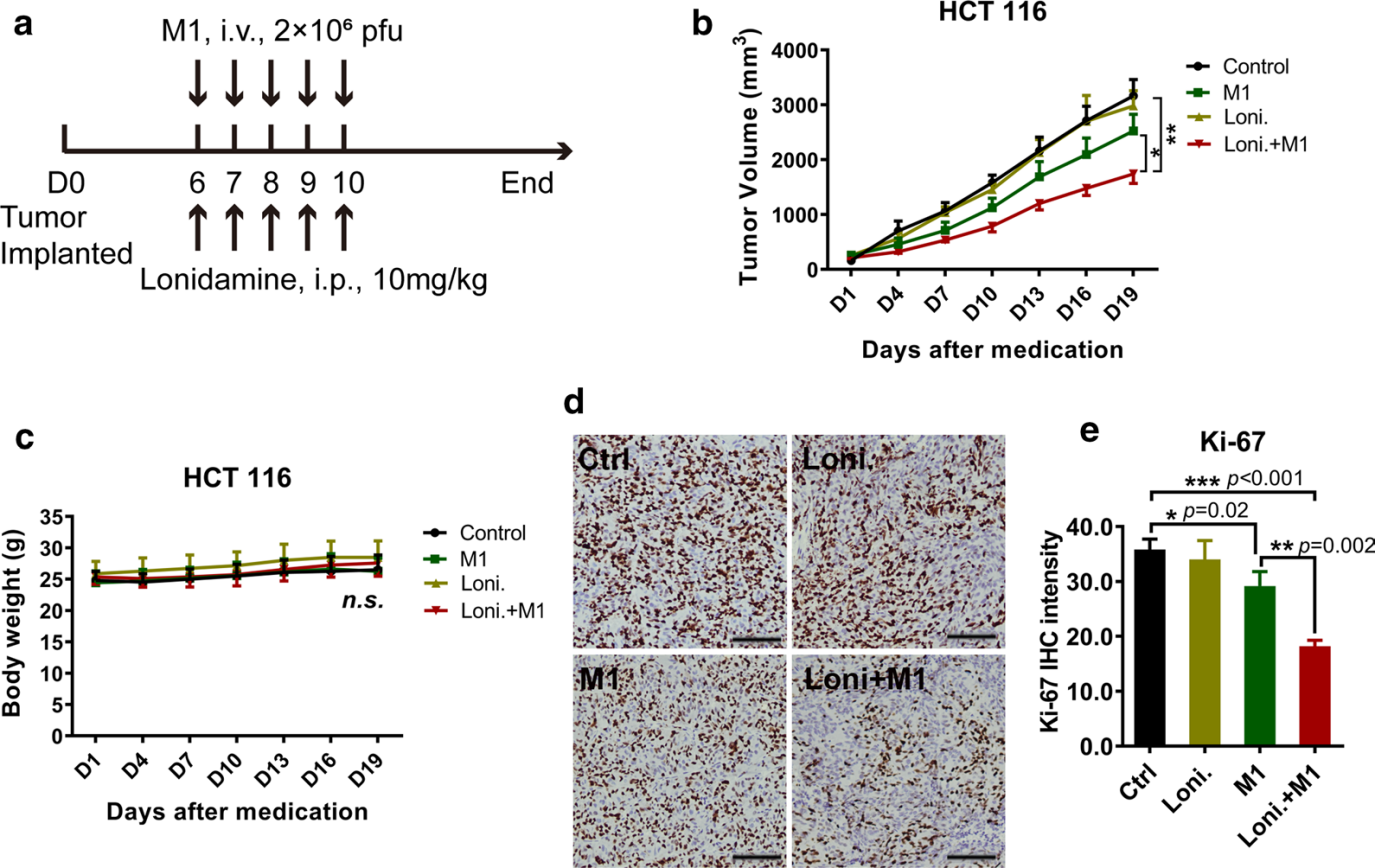
Lonidamine synergizes with M1 virus to induce tumor regression in vivo*.* **a** Schematic of the in vivo experiment. In brief, HCT 116 tumor cells were inoculated in the hind flanks of nude mice. Six days later, tumors were visible, and the mice were randomly grouped and treated for seven days with different agents: control, M1 virus (i.v., 2 × 10^6^ pfu/animal), lonidamine (i.p., 10 mg/kg/animal), and lonidamine plus M1 virus. Tumors were measured every other day, and the tumor volumes were calculated by the following formula: (length × width^2^)/2. n = 6. **b**, **c** Tumor volume and mouse weight curves. Statistical analysis was performed by repeated measures ANOVA. **d**, **e** At the experimental endpoint, tumors in each group were excised from mice, the expression of Ki-67 in tumors was detected by IHC staining, and the IHC staining intensity was analyzed with ImageScope. n = 3, Scale bars, 100 μm. Statistical analysis was performed by one-way ANOVA with Dunnett’s tests for pairwise comparisons. The error bars indicate the mean ± SD values from different mice. *ns* nonsignificant; *p < 0.05, **p < 0.01, ***p < 0.001

To investigate whether lonidamine potentiates the oncolytic effect of M1 virus in vivo, a xenograft tumor model was established with HCT 116 tumor cells in nude mice, which were then treated with control, lonidamine, M1 virus, or lonidamine plus M1 virus (Fig. 4a). Intravenous injection of M1 virus slightly inhibited tumor growth. However, compared to the other three treatments, the combination of lonidamine and M1 virus resulted in reduced tumor growth, therefore inhibiting tumor progression (Fig. 4b). Moreover, none of the treatments affected the weight of the mice (Fig. 4c), indicating the potential safety of the combination therapy. Furthermore, immunohistochemical (IHC) staining was performed on tumor tissues to detect the expression of Ki-67 in order to assess proliferation. Ki-67 was correspondingly downregulated (Fig. 4d, e) in the combination treatment group. These results suggest that the combination of lonidamine and M1 virus is an appropriate strategy for treating cancer in vivo.

## Discussion

This research first demonstrated the crosstalk between glycolysis and the replication of the oncolytic virus M1. M1 virus activates glycolytic flux in host tumor cells, while hijacking glycolysis prevents M1 virus infection and subsequent tumor cell lysis. This process conforms to the demand for energy and core biomolecules necessary for successful virion synthesis [12]. Virus-induced metabolic reprogramming mirrors the Warburg effect in cancer, which is a glycolytic metabolism signature that has been fully developed as an antitumor target [21]. However, our research suggested that M1 virus should not be combined with antitumor agents targeting the Warburg effect in the clinic. In addition, these antitumor agents could be developed as risk control drugs to abolish the undiscovered adverse effects of M1 virus in cancer patients in the future. On the other hand, lonidamine could be developed as a synergist for oncolytic virus M1. Moreover, our research indicates the possibility of combination therapy with lonidamine and other oncolytic viruses.

Lonidamine interferes with glycolysis in tumor cells by inhibiting hexokinase 2 (HK2), the first rate-limiting enzyme of glycolysis. However, subsequent studies have revealed additional pharmacological targets of this drug, such as voltage-dependent anion channel (VDAC) in the outer mitochondrial membrane (OMM); respiratory complex II, also called succinate dehydrogenase (SDH); the mitochondrial pyruvate carrier (MPC); and the H^+^-coupled monocarboxylate transporters MCT1 and MCT4. Here, we reported that lonidamine potentiates the oncolytic effect of oncolytic virus M1 and does not function by targeting HK2. Previous studies and our study indicate that the anticancer effects of lonidamine do not occur via a single target, and the actual target of lonidamine when used in combination with oncolytic viruses remains unclear. The apparent lack of specificity for the interaction of lonidamine with its targets is also intriguing. Answering these questions might help to determine the pharmacological mechanisms and to guide the clinical study of lonidamine monotherapy or combination therapy [22].

In this study, we found that lonidamine is involved in multiple biological processes, such as inhibition of antiviral genes, including MYC, and potentiation of M1 virus-mediated ER stress and caspase activity. Thus, determining the mechanism by which hexokinase inhibitors controls the above phenomenon is very important. Among these biological processes, the key events are the inhibition of antiviral signaling and the subsequent increase in M1 viral replication. Potentiation of ER stress and caspase activity-mediated apoptosis might be the accompanying events induced by increased M1 virus infection. Although the actual target of lonidamine when used in combination with M1 virus is unclear, it can be deduced that lonidamine might affect the glycolytic metabolism process, as suggested by previous reports [22]. Genes controlling glycolytic metabolism can also regulate the antiviral response [12, 23]; thus, lonidamine might inhibit the antiviral response by targeting related glycolytic metabolism genes. However, further investigation is needed to determine the specific target of lonidamine.

The tumor selectivity of oncolytic viruses is largely conferred by tumor-specific aberrations in signaling pathways that normally sense and block viral replication. Research has thoroughly established that cancer-specific aberrations in *BCL-2*, *WNT*, *EGFR*, *RAS*, *TP53*, *RB1*, *PTEN* and other cancer-related genes predispose cancer cells to viral infection [5, 24, 25]. For example, Newcastle disease virus (NDV) targets cancer cells overexpressing *BCL-XL,* which prevents apoptosis and thereby permits the virus to utilize the cellular transcription and translation machinery to synthesize viral nucleocapsids [26]. Cancer cells with *RAS* mutations cannot activate the PKR pathway, which functions to prevent viral production and spread, rendering cancer cells permissive to infection with reovirus, herpesvirus and vaccinia virus [27–30]. We previously identified that zinc finger antiviral protein (ZAP) deficiency mediates the tumor selectivity of M1 virus [7]; however, the relationship between the tumor selectivity of M1 virus and oncogenic signals has not yet been illuminated. Here, we demonstrated that lonidamine enhances the infection and tumoricidal effect of M1 virus by inhibiting MYC, a commonly overactivated oncogene. These results suggest that MYC could be a selective biomarker and that a low MYC level indicates the oncolytic efficiency of M1 virus.

## Conclusions

In summary, we demonstrated that lonidamine enhances the oncolytic effect of M1 virus in a manner independent of hexokinase 2 but instead by inhibiting antiviral immunity and that MYC deficiency is a potential selective biomarker for the oncolytic efficiency of M1 virus.

## Supplementary information

**Additional file 1.** Lonidamine inhibits the activity of hexokinase 2 in vitro and in HCT 116 cells.

**Additional file 2.** Knock down of HK2 inhibited the infection rate of M1 virus in HCT-8 cells.

**Additional file 3.** Lonidamine attenuates the IFN-β production induced by M1 virus but does not affects the IFN-α production.

**Additional file 4.** The efficiency of siRNAs to MYC, SECTIM1, and ADAM11 in HCT 116 cell line.

## Data Availability

The microarray data was submitted to the Gene Expression Omnibus (GEO) (https://www.ncbi.nlm.nih.gov/geo/). The accession number is GSE152876. The datasets used and/or analysed during the current study are available from the corresponding author on reasonable request.
