# Supplementary material for: Lonidamine potentiates the oncolytic efficiency of M1 virus independent of hexokinase 2 but via inhibition of antiviral immunity
Source: Cancer Cell Int. 2020 Nov 2;20:532. doi: 10.1186/s12935-020-01598-w (PMC7607643; doi:10.1186/s12935-020-01598-w)
Supplement: Supplementary file 1 — Additional file 1. Lonidamine inhibits the activity of hexokinase 2 in vitro and in HCT 116 cells. [file 12935_2020_1598_MOESM1_ESM.docx]

**Additional information**


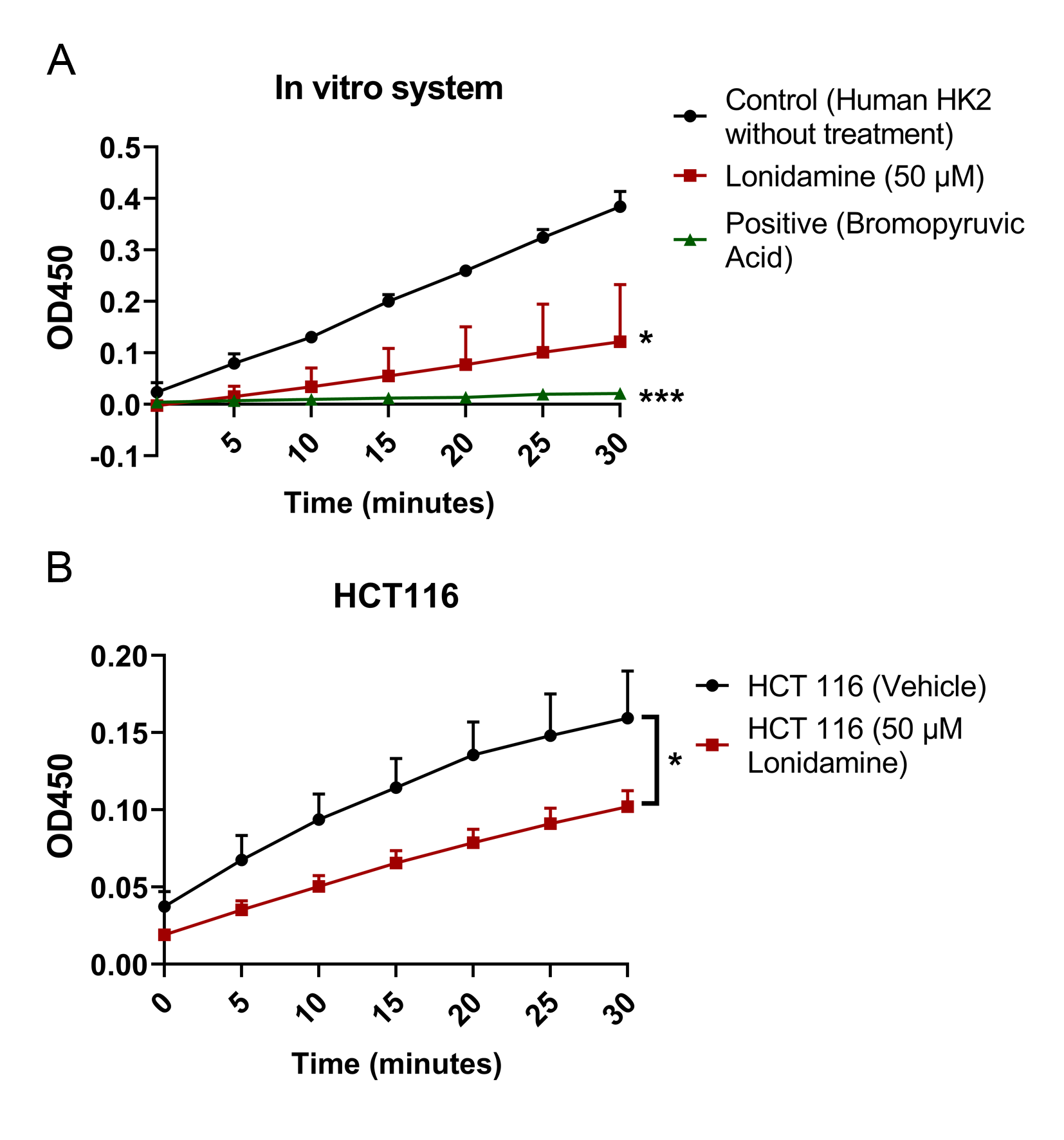


**Additional file 1. Lonidamine inhibits the activity of hexokinase 2 *in vitro* and in HCT 116 cells.**

A, The inhibition of hexokinase 2 activity by lonidamine in a test tube was detected by the Hexokinase II Inhibitor Screening Kit (Colorimetric) according to the instruction. Bromopyruvic Acid was used as a positive control. n=3. Statistical analysis was performed by repeated measures of ANOVA. B, The inhibition of hexokinase activity by lonidamine in HCT 116 cells was detected by the Hexokinase II Inhibitor Screening Kit (Colorimetric) according to the instruction. HCT 116 cellular protein lysates were used as HK2 enzyme for the reaction. n=3. Statistical analysis was performed by repeated measures of ANOVA. The error bars indicate the mean ± SD values from three independent experiments. *, *p*<0.05; *** *p*<0.001.
