# Supplementary material for: Lonidamine potentiates the oncolytic efficiency of M1 virus independent of hexokinase 2 but via inhibition of antiviral immunity
Source: Cancer Cell Int. 2020 Nov 2;20:532. doi: 10.1186/s12935-020-01598-w (PMC7607643; doi:10.1186/s12935-020-01598-w)
Supplement: Supplementary file 2 — Additional file 2. Knock down of HK2 inhibited the infection rate of M1 virus in HCT-8 cells. [file 12935_2020_1598_MOESM2_ESM.docx]

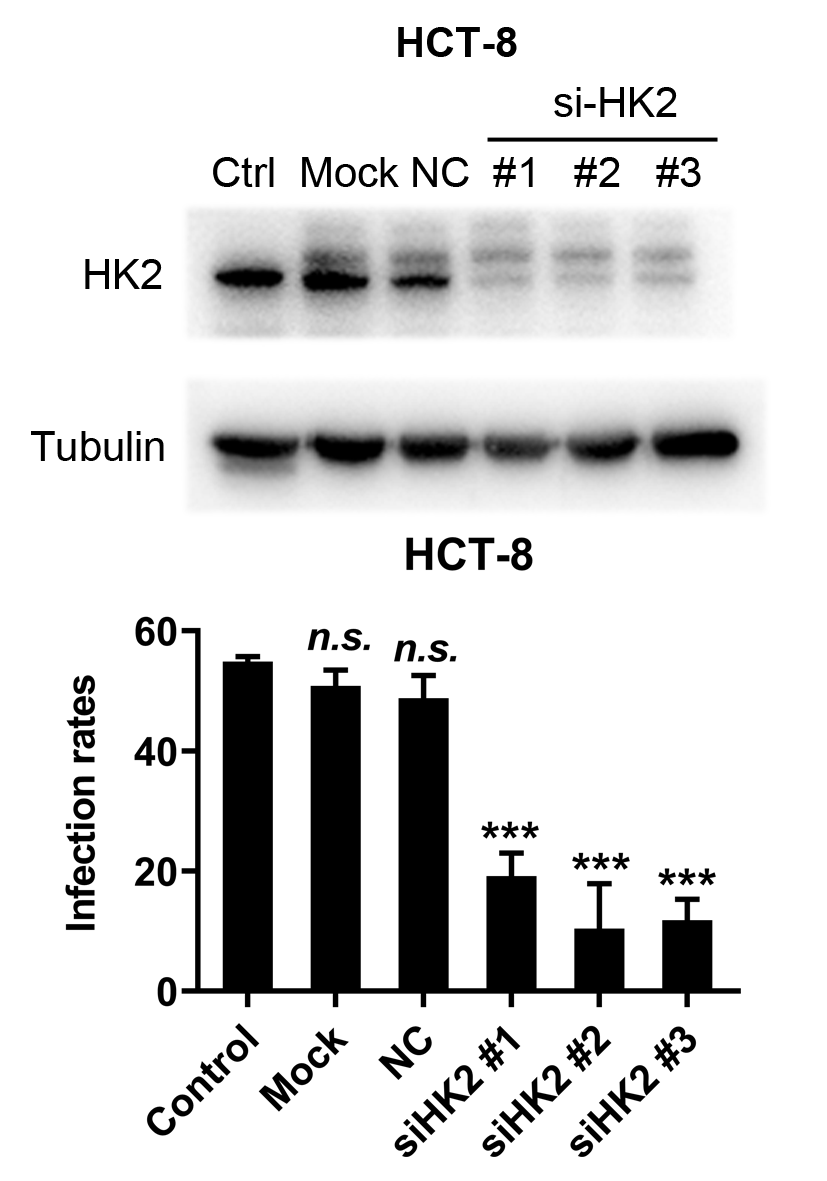


**Additional file 2. Knock down of HK2 inhibited the infection rate of M1 virus in HCT-8 cells.**

The HCT-8 cell line was treated with siRNAs targeting HK2 for 48 hours. M1 virus (MOI=1 pfu/cell) was added for another 24 hours, and the infection rate of M1 virus (GFP percentage) was then determined by flow cytometry. n=3. Statistical analysis was performed by one-way ANOVA with Dunnett’s test for pairwise comparisons. The knockdown efficiency of the siRNAs (48 hours) targeting HK2 in the HCT 116 cell line was determined by western blotting. The error bars indicate the mean ± SD values from three independent experiments. *n.s.*, nonsignificant; *** *p*<0.001.
