# Supplementary material for: Lonidamine potentiates the oncolytic efficiency of M1 virus independent of hexokinase 2 but via inhibition of antiviral immunity
Source: Cancer Cell Int. 2020 Nov 2;20:532. doi: 10.1186/s12935-020-01598-w (PMC7607643; doi:10.1186/s12935-020-01598-w)
Supplement: Supplementary file 3 — Additional file 3. Lonidamine attenuates the IFN-β production induced by M1 virus but does not affects the IFN-α production. [file 12935_2020_1598_MOESM3_ESM.docx]

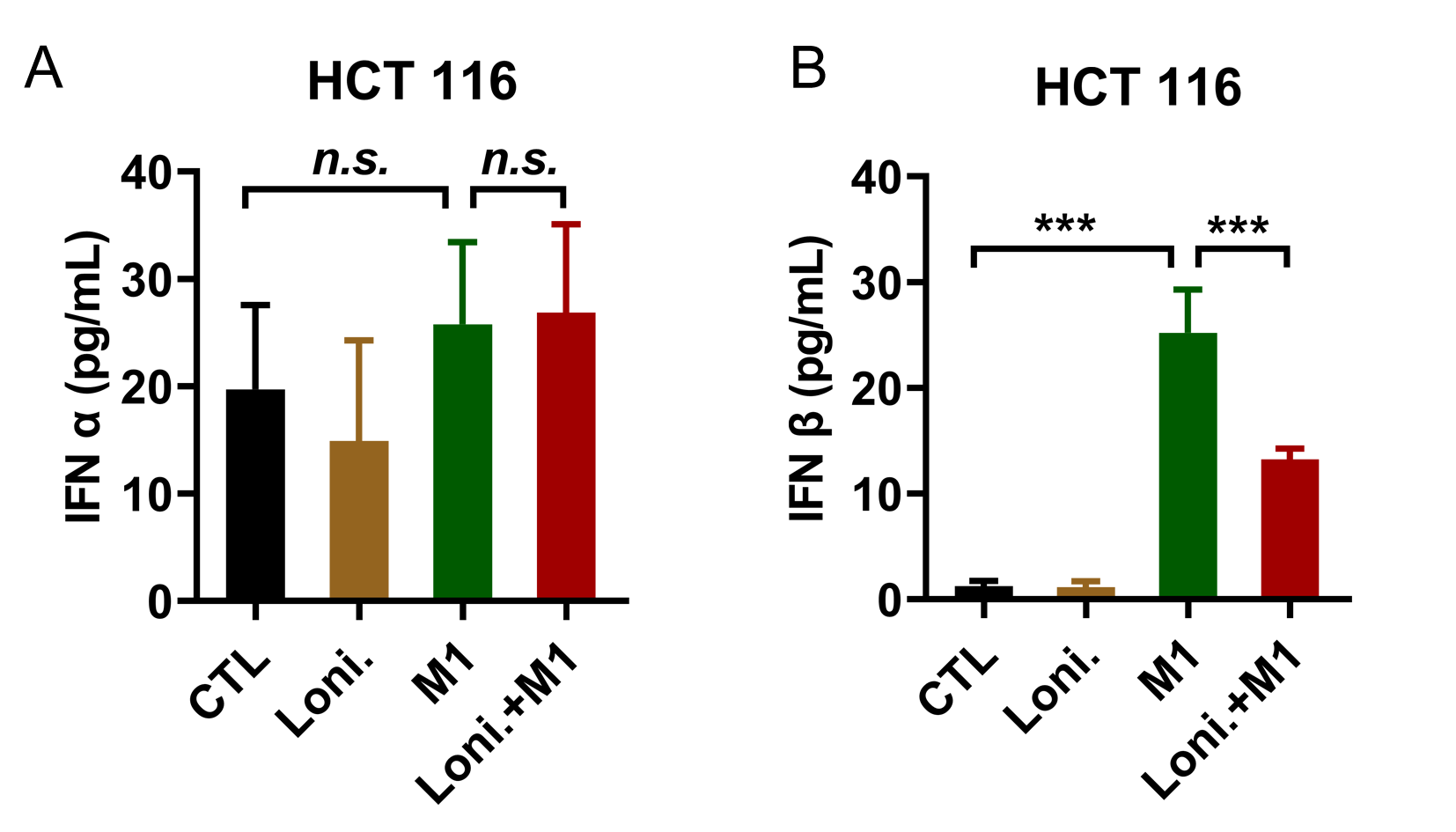


**Additional file 3. Lonidamine attenuates the IFN-β production induced by M1 virus but does not affects the IFN-α production.**

A-B, The HCT 116 cell line was treated with vehicle, lonidamine (50 μM), M1 virus (MOI=1 pfu/cell), or M1 virus plus lonidamine for 48 hours. The secretion of IFN-α and IFN-β in the supernatant was detected by ELISA. n=4. Statistical analysis was performed by one-way ANOVA with Dunnett’s test for pairwise comparisons. The error bars indicate the mean ± SD values from four independent experiments. *n.s.*, nonsignificant; *** *p*<0.001.
