# Supplementary material for: Lonidamine potentiates the oncolytic efficiency of M1 virus independent of hexokinase 2 but via inhibition of antiviral immunity
Source: Cancer Cell Int. 2020 Nov 2;20:532. doi: 10.1186/s12935-020-01598-w (PMC7607643; doi:10.1186/s12935-020-01598-w)
Supplement: Supplementary file 4 — Additional file 4. The efficiency of siRNAs to MYC, SECTIM1, and ADAM11 in HCT 116 cell line. [file 12935_2020_1598_MOESM4_ESM.docx]

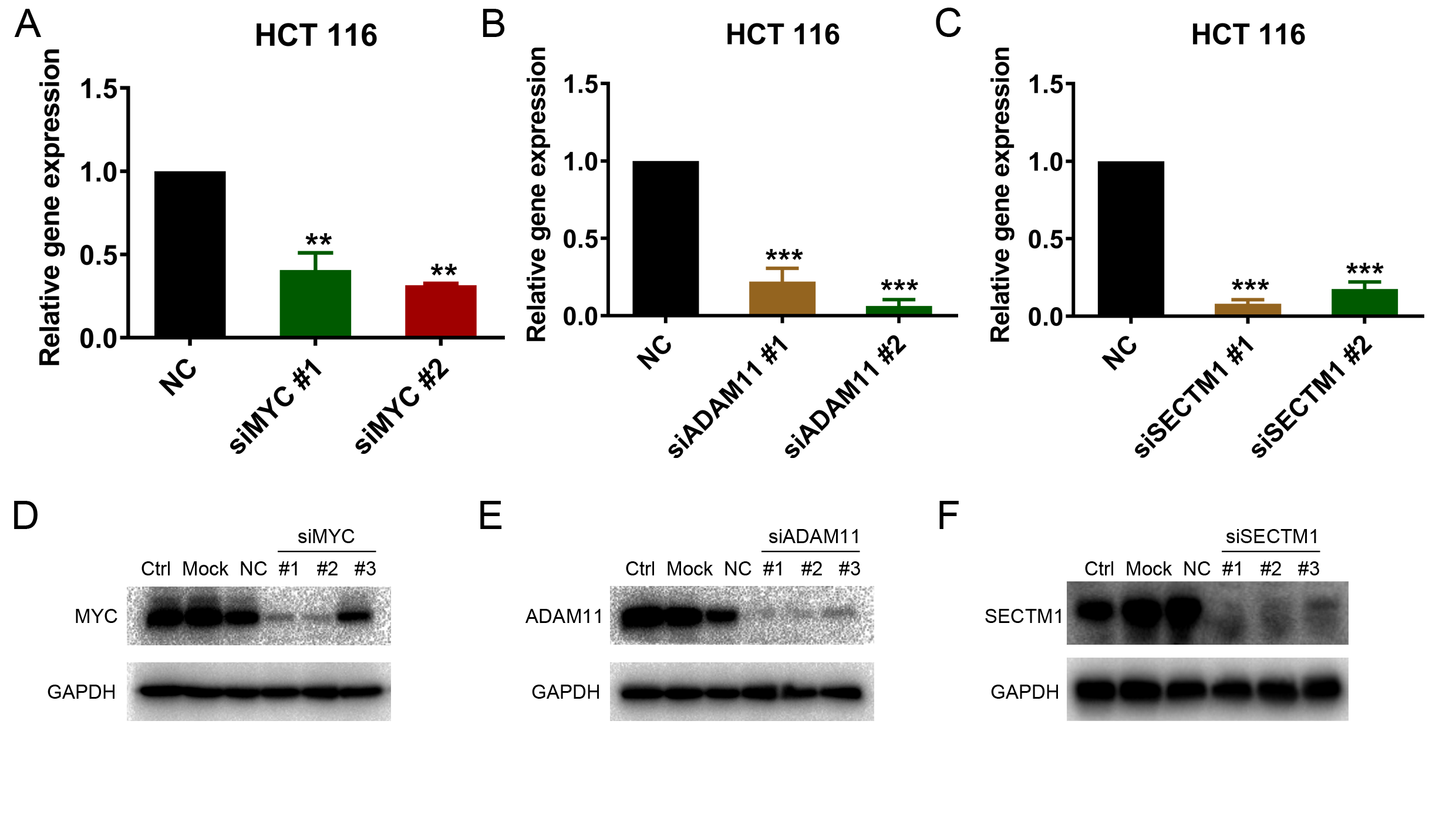


**Additional file 4. The efficiency of siRNAs to *MYC*, *SECTIM1*, and *ADAM11* in HCT 116 cell line.**

A-F, The HCT 116 cell line was treated with siRNAs targeting *ADM11*, *SECTM1*, and *MYC* for 48 hours, efficiency of siRNAs to these genes was detected by RT-qPCR and western blot. n=3. Statistical analysis was performed by one-way ANOVA with Dunnett’s test for pairwise comparisons. The error bars indicate the mean ± SD values from three independent experiments. **, *p*<0.01; *** *p*<0.001.
